# Abnormal Calcium Handling in Duchenne Muscular Dystrophy: Mechanisms and Potential Therapies

**DOI:** 10.3389/fphys.2021.647010

**Published:** 2021-04-09

**Authors:** Satvik Mareedu, Emily D. Million, Dongsheng Duan, Gopal J. Babu

**Affiliations:** ^1^Department of Cell Biology and Molecular Medicine, New Jersey Medical School, Rutgers University, Newark, NJ, United States; ^2^Department of Molecular Microbiology and Immunology, The University of Missouri, Columbia, MO, United States; ^3^Department of Biomedical, Biological & Chemical Engineering, The University of Missouri, Columbia, MO, United States

**Keywords:** Duchenne muscular dystrophy, calcium, sarco(endo)plasmic reticulum calcium ATPase, sarcolipin, ryanodine receptor, sarcolemma, dystrophin, mitochondria

## Abstract

Duchenne muscular dystrophy (DMD) is an X-linked muscle-wasting disease caused by the loss of dystrophin. DMD is associated with muscle degeneration, necrosis, inflammation, fatty replacement, and fibrosis, resulting in muscle weakness, respiratory and cardiac failure, and premature death. There is no curative treatment. Investigations on disease-causing mechanisms offer an opportunity to identify new therapeutic targets to treat DMD. An abnormal elevation of the intracellular calcium (${\text{Ca}}_{\text{i}}^{2+}$) concentration in the dystrophin-deficient muscle is a major secondary event, which contributes to disease progression in DMD. Emerging studies have suggested that targeting Ca^2+^-handling proteins and/or mechanisms could be a promising therapeutic strategy for DMD. Here, we provide an updated overview of the mechanistic roles the sarcolemma, sarcoplasmic/endoplasmic reticulum, and mitochondria play in the abnormal and sustained elevation of ${\text{Ca}}_{\text{i}}^{2+}$ levels and their involvement in DMD pathogenesis. We also discuss current approaches aimed at restoring Ca^2+^ homeostasis as potential therapies for DMD.

## Introduction

Duchenne muscular dystrophy (DMD) is X-linked and is the most common form of muscle wasting disease. It affects 1 in 3,500 to 5,000 male births (Mendell and Lloyd-Puryear, 2013). DMD is caused by mutations in the dystrophin gene, which leads to the loss of a functional dystrophin protein (Monaco et al., 1986). DMD is characterized by progressive loss of muscle mass and function due to muscle degeneration, necrosis, and fatty fibrosis, resulting in wheelchair dependence (Ervasti and Campbell, 1993; Bushby et al., 2010; Connolly et al., 2013; Aartsma-Rus et al., 2016; Duan et al., 2021). Eventually, patients die of respiratory failure and/or cardiomyopathy (Finsterer, 2006). There is currently no curative treatment for DMD. Restoring dystrophin function via gene replacement or repair therapy is attractive and promising. Although there is a favorable outcome in preclinical studies, the immunogenicity of the newly expressed dystrophin protein remains a concern. In addition, the complexity of thousands of disease-causing mutations in the dystrophin gene creates challenges for gene repair therapy. Alternatively, targeting major disease-causing mechanisms offers an opportunity to treat all DMD patients without the complications of dystrophin replacement or repair therapies.

Dystrophin is a rod-shaped cytoskeletal protein, primarily expressed in muscles. Dystrophin links the intracellular cytoskeleton network to the transmembrane components of the dystrophin–glycoprotein complex (Gao and McNally, 2015; Allen et al., 2016). Growing evidence suggests dystrophin in addition to maintaining the structural integrity of the sarcolemma plays a key role in regulating signaling pathways. This includes the nitric oxide pathway, Ca^2+^ entry, and the production of reactive oxygen species (ROS). In the absence of dystrophin, these pathways are damaged and contribute to muscle pathology. For details on these pathways and their clinical implications in DMD, refer to a recent review by Allen et al. (2016).

Among the various disease-causing mechanisms, changes in intracellular calcium (${\text{Ca}}_{\text{i}}^{2+}$) levels in dystrophin-deficient muscle fibers have been studied for many years. An increase in both Ca^2+^ influx and cytosolic Ca^2+^ concentration has been reported in *mdx* myofibers (Turner et al., 1988; Hopf et al., 1996). Although many studies have shown higher levels of Ca^2+^ content in the muscle fibers from *mdx* mice, others have reported no significant rise in Ca^2+^ concentration (Gillis, 1996, 1999; Gailly, 2002; Whitehead et al., 2006). Dystrophin is indispensable for maintaining the structural integrity of the striated muscle cell (Petrof et al., 1993). Thus, lack of dystrophin destabilizes sarcolemma integrity, making the sarcolemma more susceptible to contraction-induced damage. This leads to myonecrosis (necrosis of myofibers), which in turn stimulates fiber regeneration. In *mdx* mice, the onset of myonecrosis starts at 2 weeks of age. Myonecrosis peaks between 3 and 4 weeks of age and is significantly decreased and stabilized by 6 to 8 weeks of age. Eventually, ~80% of myofibers in adult *mdx* mice are regenerated fibers (Grounds et al., 2008). A sharp increase in total Ca^2+^ content has been shown at the peak of myonecrosis. The total Ca^2+^ content returns to normal level in later stages (Reeve et al., 1997). Studies from Head's laboratory suggest that regenerated fibers display branched morphology. It has been hypothesized that myofiber branching rather than dystrophin deficiency increases muscle susceptibility to damage (Head, 2010; Chan and Head, 2011). These studies further suggest that the branching of fibers resulted in altered ion channel function and excessive Ca^2+^ influx, which further exacerbates muscle damage. Muscle damage and degeneration are therefore suggested to play an important role in stress-induced membrane tears and dysfunction of sarcolemmal ion channels that lead to abnormal Ca^2+^ influx from the extracellular matrix (ECM) to the cytosol (Turner et al., 1988, 1991; Moens et al., 1993; Alderton and Steinhardt, 2000; Burr and Molkentin, 2015). In addition, there is a defect in Ca^2+^ cycling between the cytosol and the sarcoplasmic reticulum (SR)/endoplasmic reticulum (ER), a major internal Ca^2+^ store in striated muscles. These changes result in a chronic accumulation of Ca^2+^ in the cytoplasm. Thus, the lack of dystrophin results in muscle degeneration and improper regeneration, which may contribute to abnormal ${\text{Ca}}_{\text{i}}^{2+}$ handling.

Several lines of evidence suggest that sustained elevation of cytosolic Ca^2+^ levels underlies muscle pathology and dysfunction in DMD. First, increased cytosolic Ca^2+^ levels enhance the expression and activity of calpains, the Ca^2+^ dependent proteases in dystrophic muscles (Spencer et al., 1995; Hussain et al., 2000; Shanmuga Sundaram et al., 2006; Voit et al., 2017). Calpain activation results in proteolytic damage to cellular proteins and the myofibrillar network (Dayton et al., 1976; MacLennan et al., 1991; Bartoli and Richard, 2005). In support of this notion, treatment with BN 82270, a membrane-permeable calpain inhibitor, improves muscle function in *mdx* mice (Burdi et al., 2006). Studies from our laboratory have shown normalization of ${\text{Ca}}_{\text{i}}^{2+}$ cycling by improving SR Ca^2+^ ATPase (SERCA) activity decreases calpain activity and improves muscle function in dystrophin and utrophin double-mutant (*mdx:utr*^−/−^) mice (Voit et al., 2017). In addition to calpains, increased cytosolic Ca^2+^ levels also activate phospholipase A, which digests cellular membranes such as the sarcolemma (Lindahl et al., 1995). Second, the sustained elevation of ${\text{Ca}}_{\text{i}}^{2+}$ concentration activates apoptotic and necrotic cell death pathways in DMD (Tidball et al., 1995; Morgan et al., 2018). Third, abnormal ${\text{Ca}}_{\text{i}}^{2+}$ concentration may influence muscle differentiation and compromise muscle regeneration. In this regard, we have recently demonstrated improving ${\text{Ca}}_{\text{i}}^{2+}$ cycling in dystrophic myoblasts improves myoblast fusion and differentiation (Niranjan et al., 2019). Fourth, rapid change in free Ca^2+^ levels in the cytoplasm is essential for proper initiation of muscle contraction and relaxation (Calderon et al., 2014). Thus, chronic accumulation of Ca^2+^ in the cytosol can affect muscle function in DMD. Fifth, cytoplasmic Ca^2+^ levels influence mitochondrial Ca^2+^ uptake, which in turn leads to altered metabolism and increased production of ROS. The failure to mitigate supraphysiological levels of oxygen radicals results in the loss of membrane potential and cell death (Feno et al., 2019). Collectively, abnormal ${\text{Ca}}_{\text{i}}^{2+}$ cycling plays a pivotal role in DMD pathogenesis, and restoration of ${\text{Ca}}_{\text{i}}^{2+}$ homeostasis may ameliorate muscle disease and cardiomyopathy in DMD. A better understanding of the molecular mechanisms underlying abnormal ${\text{Ca}}_{\text{i}}^{2+}$ cycling will aid in identifying novel therapeutic targets for DMD. Below, we review Ca^2+^ handling in normal muscle and how changes at the sarcolemma, SR, and mitochondria cause Ca^2+^ dysregulation in DMD. We also highlight the current studies on improving SERCA function as a strategy to mitigate skeletal muscle disease and cardiomyopathy in DMD.

## Calcium Handling in Normal Muscle

**Figure 1:**
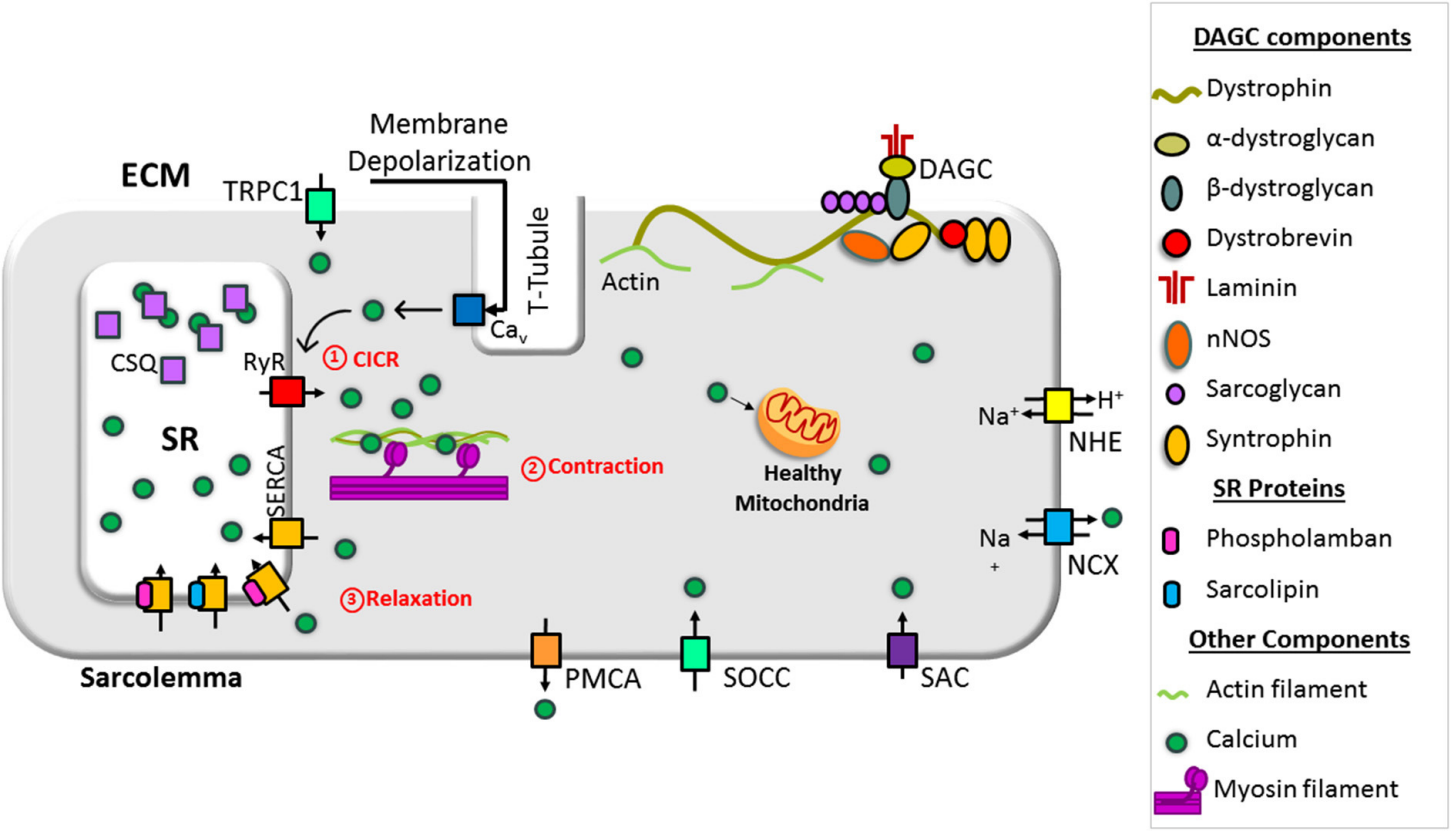
Schematic representation of intracellular Ca^2+^ cycling in a normal muscle cell. Dystrophin stabilizes muscle cells by linking the cytoskeleton (F-actin, intermediate filaments, microtubule) to the extracellular matrix via the dystrophin-associated glycoprotein complex (DAGC). Ca^2+^-induced Ca^2+^ release (CICR) occurs through activation of voltage-gated, L-type Ca^2+^ channels (Ca_v_) and the release of Ca^2+^ from the sarcoplasmic/endoplasmic reticulum (SR) via ryanodine receptor (RyR). CICR activates muscle contraction. Resequestration of Ca^2+^ back into the SR by SR Ca^2+^ ATPase (SERCA) initiates muscle relaxation. The physiological intracellular Ca^2+^ level regulates mitochondrial Ca^2+^ content and function and maintains cellular energetics. DG, dystroglycan; NCX, sodium–calcium exchanger; NHE, sodium–proton exchanger; PMCA, plasma membrane Ca^2+^ ATPase; SAC, stretch-activated channels; SOCC, store-operated Ca^2+^ channel; TRPC1, transient receptor potential channel 1.

Contraction and relaxation cycles are controlled voluntarily in skeletal muscle and involuntarily in cardiac muscle and are tied with Ca^2+^ cycling between the SR and cytoplasm. Ca^2+^ cycling in normal muscle is depicted in Figure 1. In muscles, extracellular Ca^2+^ concentrations are ~2–4 mM, and resting cytosolic concentrations are ~50–250 nM. SR Ca^2+^ concentrations are ~0.4–0.5 mM (MacLennan and Kranias, 2003; Gehlert et al., 2015; Kuo and Ehrlich, 2015). In the heart, the resting diastolic Ca^2+^ levels are ~100 nM and during systole, cytosolic Ca^2+^ levels increase to ~1 μM (MacLennan and Kranias, 2003). Excitation and contraction (EC) coupling in skeletal and cardiac muscles shares many similarities. In both muscles, the action potential activates voltage-gated, L-type Ca^2+^ channels (Ca_v_1.1 in skeletal muscles and Ca_v_1.2 in cardiac muscle) in the sarcolemma, which promotes Ca^2+^ release from the SR via the ryanodine receptor (RyR1 in skeletal muscles and RyR2 in cardiac muscle). This process is known as Ca^2+^-induced Ca^2+^ release (CICR) (MacLennan and Kranias, 2003; Rios, 2018). Although CICR was first discovered in skeletal muscle, it does not play a major role in skeletal muscle contraction (Lamb, 2000; Endo, 2009; Rios, 2018). In cardiac muscle, the interaction between Ca_v_1.2 and RyR2 depends on CICR, whereas in skeletal muscle, Ca_v_1.1 and RyR1 physically interact. This interaction is independent of the Ca^2+^ influx via Ca_v_1.1 (Protasi, 2002; Franzini-Armstrong, 2018). It also activates the opening of RyR1 in response to Ca_v_1.1 voltage sensor activation, a process known as Ca^2+^-independent, depolarization-induced Ca^2+^ release (also called voltage-induced Ca^2+^ release) (Rios, 2018). The mechanism by which these two channels interact is not fully understood. A recent study shows junctophilins (protein localized in junctional SR) enable the Ca_v_1.1 to efficiently couple with RyRs through protein–protein interaction. This mechanism is considered crucial for efficient EC coupling in adult skeletal muscles (Nakada et al., 2018). Studies have also shown the importance of store-operated Ca^2+^ entry independent of Ca_v_1.1 in the regulation of skeletal muscle contraction (Avila-Medina et al., 2018). Store-operated Ca^2+^ entry machinery located in the triad of skeletal muscle contains many proteins including stromal interaction molecule 1 (STIM1), Orai, transient receptor potential canonical (TRPC) channel, and RyRs. These proteins are involved in Ca^2+^ store restoration during the intense work of skeletal muscles (Pan et al., 2014; Avila-Medina et al., 2018).

Both in skeletal and cardiac muscles, Ca^2+^ released from the SR binds to troponin C, a thin filament sarcomeric protein, and initiates muscle contraction. Muscle relaxation is initiated by Ca^2+^ removal from the cytoplasm. Approximately 70–90% of Ca^2+^ is removed by SERCA (SERCA1 and SERCA2a in skeletal muscle and SERCA2a in cardiac muscle) and resequestrated into the lumen of the SR. Most of the remaining Ca^2+^ is extruded out of the cell via sarcolemmal Ca^2+^ transport proteins. These include the sodium (Na^+^)–Ca^2+^ exchanger (NCX) and plasma membrane Ca^2+^ ATPase. A small amount of Ca^2+^ is taken up by the mitochondria via the mitochondrial uniporter (MCU). Thus, SR Ca^2+^ cycling is not only important for muscle contraction and relaxation cycle but also helps to maintain cytosolic Ca^2+^ levels. For extensive reviews on Ca^2+^ handling in normal skeletal muscle and heart, refer to the recent reviews (Endo, 2009; Lee, 2010; Eisner et al., 2017; Avila et al., 2019).

## Sarcolemmal Contribution to Abnormal ${\text{Ca}}_{\text{i}}^{2+}$ Handling in Dystrophic Muscles

**Figure 2:**
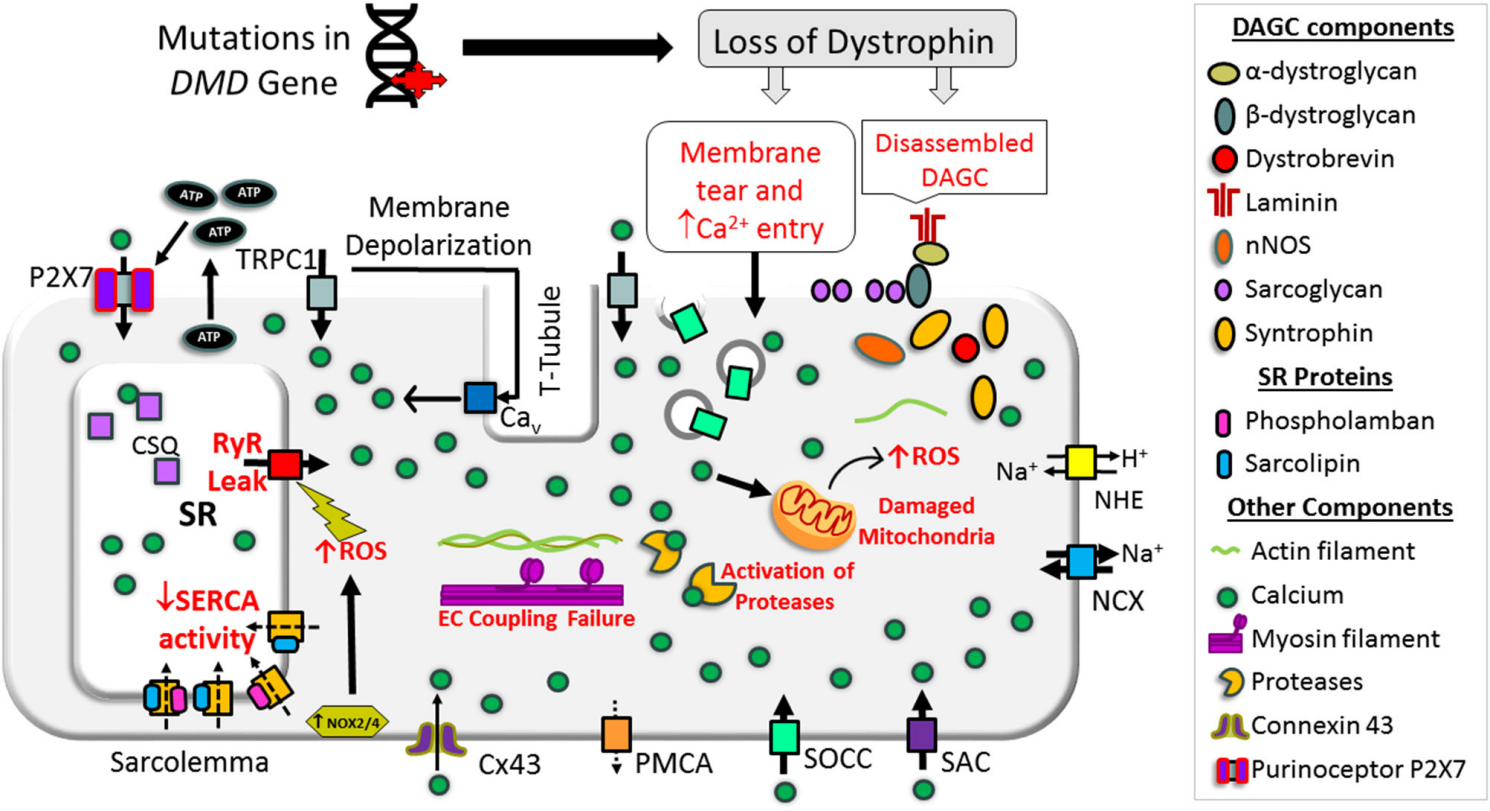
Schematic representation of mechanisms causing ${\text{Ca}}_{\text{i}}^{2+}$ overload in a dystrophin-deficient muscle cell. Loss of dystrophin causes destabilization of dystrophin-associated glycoprotein complex (DAGC), membrane tear, and activation of store-operated calcium channels (SOCCs), resulting in abnormal Ca^2+^ entry. High concentrations of extracellular ATP can activate abnormal Ca^2+^ influx via P2X7. RyR dysfunction causes Ca^2+^ leak from the SR and SERCA dysfunction compromises Ca^2+^ resequestration. NOX activation can also increase RyR Ca^2+^ leak. These changes result in abnormal and chronic elevation of the cytoplasmic Ca^2+^ levels. Supraphysiological level Ca^2+^ activates Ca^2+^-dependent proteases and phospholipase and causes muscle necrosis and replacement of muscle by fatty and fibrotic tissues. Sustained elevation of cytoplasmic Ca^2+^ levels also affects mitochondrial function and increases reactive oxygen species (ROS). Collectively, these changes lead to muscle wasting and contractile dysfunction. Bold and broken arrows indicate the enhanced and decreased function of the Ca^2+^ channels, respectively. Cx43, connexin 43; NCX, sodium–calcium exchanger; NHE, sodium–proton exchanger; NOX, NADPH oxidases; PMCA, plasma membrane Ca^2+^ ATPase; P2X7, P2X purinoceptor 7; SAC, stretch-activated channels; SOCC, store-operated Ca^2+^ channel; TRPC1, transient receptor potential channel 1.

In dystrophin-deficient muscle, the function of sarcolemmal Ca^2+^ channels is altered and contributes to the abnormal elevation of cytoplasmic Ca^2+^ concentration. Schematic representation of abnormal Ca^2+^ handling via the sarcolemma in dystrophin-deficient muscle is shown in Figure 2.

### Ca^2+^ Entry Through Membrane Tearing

A primary function of dystrophin is to link the cytoskeleton with the ECM through direct or indirect interaction with the proteins in the dystrophin-associated glycoprotein complex (DAGC) and direct interaction with actin microfilaments, intermediate filaments, and microtubules (Koenig et al., 1988; Ervasti and Campbell, 1993; Petrof et al., 1993; Chakkalakal et al., 2005; Gao and McNally, 2015). The absence of dystrophin disrupts the DAGC and makes the sarcolemma more susceptible to microtears during mechanical stress (Petrof et al., 1993; Danialou et al., 2001). Increased sarcolemmal membrane permeability is an early feature of patients affected by DMD, allowing cytosolic contents such as creatine kinase to exit skeletal muscle fibers and ions such as Ca^2+^ to enter. On the other hand, in *mdx* mice, blocking NADPH oxidase 2 (NOX2), the main ROS producer, saves muscle from force loss following eccentric contraction (Olthoff et al., 2018). Similarly, *mdx* muscle fibers repair their sarcolemma just as efficiently as wild-type fibers following laser-induced focal damage, suggesting membrane tears of this kind are sealed promptly (Cooper and Head, 2015). Thus, it is now believed that membrane tear is not a primary pathway for Ca^2+^ entry in DMD (Yeung et al., 2005; Allen and Whitehead, 2011; Cooper and Head, 2015; Olthoff et al., 2018).

### Ca^2+^ Entry Through Membrane Repair

Calcium signaling plays an important role in sarcolemma repair (Andrews et al., 2014; Cooper and Head, 2015). Increased cytosolic Ca^2+^ levels trigger Ca^2+^-dependent repair mechanisms in which exocytic vesicles patch sarcolemmal tears. This may result in increased activity of Ca^2+^ leak channels because an antagonist of these channels reduced the higher levels of hydrolysis in dystrophic myotubes to nearly normal levels (Alderton and Steinhardt, 2000). Recent studies have demonstrated the involvement of transient receptor potential mucolipin 1 (TRPML1), a lysosomal Ca^2+^ release channel needed for lysosomal exocytosis in membrane repair in DMD (Cheng et al., 2014; Yu et al., 2020). In mice, genetic ablation of TRPML1 resulted in DMD-like phenotype with an impairment in the membrane sealing in skeletal muscles (Cheng et al., 2014). On the other hand, transgenic overexpression or pharmacological activation of TRPML1 *in vivo* facilitates sarcolemma repair and alleviates dystrophic phenotypes in both skeletal and cardiac muscles of *mdx* mice (Yu et al., 2020). These studies further show that TRPML1 activation increased lysosomal biogenesis by activating the transcription factor EB and thereby facilitated sarcolemma repair to reduce muscle damage in *mdx* mice (Yu et al., 2020). Therefore, targeting lysosomal Ca^2+^ channels may represent a promising approach to treat DMD and related muscle diseases.

### Ca^2+^ Entry Through Store-Operated Calcium Channels

Store-operated calcium channels (SOCCs) are Ca^2+^ channels residing at the sarcolemma that open in response to decreased SR Ca^2+^ concentrations. The activity of SOCCs is enhanced in dystrophic muscle cells, which further contributes to the abnormal elevation of ${\text{Ca}}_{\text{i}}^{2+}$ levels in dystrophic muscles [40]. Two proteins have been identified as necessary players involved in SOCE: STIM, an ER-located Ca^2+^ sensor, and Orai, a highly Ca^2+^ selective ion channel protein in the plasma membrane (Derler et al., 2016). In dystrophin-deficient myoblasts and muscle fibers, the expression of SOCC proteins is increased (Edwards et al., 2010; Andrews et al., 2014). Interestingly, SOCCs can also be activated through a Ca^2+^-independent pathway. Specifically, Ca^2+^-independent phospholipase A_2_ (iPLA_2_) acts as an intracellular messenger and triggers Ca^2+^ entry through SOCCs (Smani et al., 2003). An elevation in iPLA_2_ levels observed in dystrophic muscles (Smani et al., 2003, 2004; Boittin et al., 2010) supports the activation of this pathway.

### Ca^2+^ Entry Through the Transient Receptor Potential Canonical Channels

TRPCs are a family of plasma membrane cation channels opened by Ca^2+^ store depletion and/or membrane stretch. In dystrophin-deficient muscles, evidence shows Ca^2+^ enters via eccentric contraction-activated stretch channels (Yeung et al., 2005). Several lines of evidence suggest the opening of TRPCs could be the main contributor of Ca^2+^ entry through the plasma membrane. To start, overexpression of TRPC1, TRPC3, and TRPC6 has been observed in *mdx* muscle (Vandebrouck et al., 2007; Gervasio et al., 2008; Boittin et al., 2010; Matsumura et al., 2011; Miyatake et al., 2016). The most widely studied of these is TRPC1. In the *mdx* muscle, TRPC1 forms a Ca^2+^ influx pathway with tyrosine–protein kinase Src and caveolin-3 (Gervasio et al., 2008). TRPC1 also interacts with dystrophin and α1-syntrophin, a member of the DAGC. In one model for TRPC1 regulation, the DAGC serves as a scaffold for signaling molecules involved in the regulation of channels formed by TRPC1 and other TRPC isoforms (Sabourin et al., 2009). In the absence of dystrophin, this regulation is lost, and SOCE is increased (Vandebrouck et al., 2007). Matsumura et al. (2011) showed that the expression levels of TRPC1 correlated with the severity of muscle disease in *mdx* mice.

In addition to canonical TRPCs, the transient receptor potential vanilloid type 2 (TRPV2) channel may also play a role in increased Ca^2+^ levels. Typically, TRPV2 is localized on intracellular organelles. In dystrophin-deficient muscle, however, it translocates to the plasma membrane (Iwata et al., 2003, 2009).

### Contribution of Sodium Regulators in ${\text{Ca}}_{\text{i}}^{2+}$ Levels in DMD

In dystrophic muscle, intracellular Na^+^ levels are increased via the opening of voltage-gated Na^+^ channels (Nav1.4) (Hirn et al., 2008), Na^+^/H^+^ exchangers (NHE) type I (Iwata et al., 2007; Burr et al., 2014), and gadolinium sensitive-stretch channels (Yeung et al., 2003). In healthy muscle, the NCX removes excess Ca^2+^ from the cytosol in exchange for Na^+^. However, in the presence of excessive cytosolic Na^+^ (as in dystrophic muscle), NCX functions in a reverse mode to remove Na^+^ from the cell. In doing so, NCX moves Ca^2+^ into the cell. Interestingly, it has been shown that increased SR Ca^2+^ release in dystrophic muscle can also trigger the NCX to work in a reverse mode and thereby increase ${\text{Ca}}_{\text{i}}^{2+}$ concentrations (Deval et al., 2002).

### Role of Voltage-Gated, L-Type Ca^2+^ Channels in ${\text{Ca}}_{\text{i}}^{2+}$ Load

The activity of the cardiac L-type Ca^2+^ channel, Ca_v_1.2, determines Ca^2+^ entry in the plateau phase (phase 2) of the action potential in cardiac myocytes. In *mdx* cardiac myocytes, Ca_v_1.2 activation is significantly increased (Koenig et al., 2014). This leads to enhanced Ca^2+^ influx via Ca_v_1.2 during the action potential. It is worth mentioning that enhanced Ca_v_1.2 activities may disturb cardiac electrophysiology and thereby cause arrhythmias in DMD.

The role of Ca_v_1.1 activity in abnormal Ca^2+^ entry in dystrophic skeletal muscle is not clear. In delta-sarcoglycan (a component of DAGC)–deficient dystrophic hamsters, the administration of diltiazem, the L-type Ca^2+^ antagonist, reduces muscle Ca^2+^ content (Bhattacharya et al., 1982). This suggests a possible pathological role played by L-type Ca^2+^ channels in dystrophic muscle. In contrast, Friedrich et al. found that Ca_v_1.1 activity is significantly reduced in the fast-twitch muscles of *mdx* mice (Friedrich et al., 2004). These studies further suggest dystrophin, and the DAGC may regulate the interaction between L-type Ca^2+^ channels and RyR, which is necessary for EC coupling (Friedrich et al., 2004, 2008).

### Other Ca^2+^ Entry Mechanisms in Dystrophin-Deficient Muscle

Dystrophic muscle damage triggers the release and elevation of extracellular ATP, which activates specific ionotropic purinoreceptors, P2X7, on immune cells and subsequently contributes to chronic inflammatory and immune responses (Gorecki, 2019). In addition, high concentrations of extracellular ATP have been shown to activate abnormal Ca^2+^ influx into dystrophic muscle cells (Young et al., 2018). This increase in ${\text{Ca}}_{\text{i}}^{2+}$ is shown to be associated with increased and prolonged activation of P2X7 purinoceptors that increase the sarcolemma permeability in dystrophic muscle cells (Young et al., 2012). Degrading extracellular ATP by apyrase reduced the ${\text{Ca}}_{\text{i}}^{2+}$ levels in *mdx* fibers (Altamirano et al., 2013). Genetic and pharmacological targeting of P2X7 reduced inflammation and increased dystrophic muscle repair (Gorecki, 2019). Thus, targeting purinergic receptors can ameliorate the abnormal Ca^2+^ entry in dystrophic muscles.

Connexins (Cx) are gap junction proteins, which are important for many physiological processes including coordinated depolarization of muscle, and ion movement between muscle cells (Cea et al., 2012). Cx function as gap junction channels and hemichannels, which mediate intercellular and transmembrane signaling, respectively. Hemichannels can act as conduits for Na^+^ and Ca^2+^ entry (Cea et al., 2012). In dystrophic muscles, Cx39, Cx43, and Cx45 are found to form functional hemichannels, which are absent in normal muscle fibers (Cea et al., 2016). The *mdx* mice deficient for Cx43/Cx45 expression in skeletal muscle show reduced basal ${\text{Ca}}_{\text{i}}^{2+}$ level and necrotic phenotype (Cea et al., 2016). Cx43 levels are significantly increased and are mislocalized in the lateral sides of cardiomyocytes in mouse models of DMD (Gonzalez et al., 2018). Altered localization of Cx43 predisposed the DMD mice to cardiac arrhythmias (Gonzalez et al., 2018). A recent study shows that hypophosphorylation of Cx43 serine triplet triggers redistribution of Cx43 to the lateral sides of cardiomyocytes and contributes to the dystrophic cardiomyopathy in *mdx* mice (Himelman et al., 2020). The expression of phosphorylation mimic Cx43 in *mdx* cardiomyocytes shows improved ${\text{Ca}}_{\text{i}}^{2+}$ signaling, a reduction of NOX2/ROS production and prevention of arrhythmias (Himelman et al., 2020). Taken together, these studies indicate that Cx overexpression and lateralization contribute to abnormal ${\text{Ca}}_{\text{i}}^{2+}$ homeostasis in dystrophin-deficient cardiac and skeletal muscles.

## Role of the SR in Abnormal ${\text{Ca}}_{\text{i}}^{2+}$ Handling in Dystrophic Striated Muscles

The SR is the major internal Ca^2+^ store in striated muscles and plays a pivotal role in the regulation of EC coupling by maintaining cytoplasmic Ca^2+^ concentrations during the muscle contraction and relaxation cycle (Santulli et al., 2017). Numerous studies suggest SR Ca^2+^ cycling is compromised in dystrophic muscle (Collet et al., 1999; Woods et al., 2004; Williams and Allen, 2007a; DiFranco et al., 2008; Capote et al., 2010) (Figure 2). Below, we review the mechanisms underlying SR Ca^2+^ cycling defects in dystrophin-deficient cardiac and skeletal muscles.

### Role of RyR in Abnormal Elevation of ${\text{Ca}}_{\text{i}}^{2+}$ Levels

Ca^2+^ release from the SR occurs via RyR, a macromolecular complex. There are three RyR isoforms reported in mammals. RyR1 is primarily expressed in skeletal muscles, RyR2 is expressed predominantly in the heart, and RyR3 is found in the brain and skeletal muscles (Conti et al., 1996; Lanner et al., 2010).

The role of the SR in cytosolic Ca^2+^ rise has been studied using chemically skinned muscle fibers that have not been mechanically stressed (Divet and Huchet-Cadiou, 2002). Studies on the skinned fibers isolated from extensor digitorum longus (EDL) and soleus muscles show that the SR Ca^2+^ release following exposure to caffeine is significantly slower in *mdx* mice. These studies suggest a more pronounced SR Ca^2+^ leak in EDL and soleus muscle fibers from *mdx* mice. Consistent with these findings, Robin et al. have shown increased passive SR Ca^2+^ leak in isolated intact myofibers prepared from FDB muscles of mdx5cv mice, an alternative dystrophin-deficient mouse model (Robin et al., 2012). On the contrary, Plant and Lynch have shown that the SR Ca^2+^ leak was unaltered in skinned *mdx* fibers (Plant and Lynch, 2003). However, peak caffeine-induced Ca^2+^ release was decreased in *mdx* fibers. In addition, all the above studies show no change in SR Ca^2+^ uptake in skinned fibers from *mdx* mice. The structural and functional defects in both cardiac and skeletal muscle RyRs have been reported in the *mdx* mouse model. Recent studies have shown that the SR Ca^2+^ release mechanism is impaired in both cardiac and skeletal muscles in DMD (Bellinger et al., 2009; Fauconnier et al., 2010). In skeletal muscle, it is believed that progressive S-nitrosylation of RyR1 and depletion of calstabin 1, a critical regulatory subunit of RyR macromolecular complex, are responsible for RyR1 Ca^2+^ leak (Bellinger et al., 2009). Similarly, S-nitrosylation and calstabin 2 depletion cause RyR2 Ca^2+^ leak and contribute to sudden cardiac arrhythmias in *mdx* mice (Fauconnier et al., 2010). In addition, RyR2 phosphorylation and oxidation have been linked to RyR2-mediated Ca^2+^ leak in the *mdx* heart. These studies further show that genetic inhibition of RyR2 phosphorylation at S2808 or S2814 can reduce RyR2 oxidation, suggesting a potential interaction between these posttranslational pathways (Williams and Allen, 2007b; Shannon, 2009; Prosser et al., 2011; Wang et al., 2015).

### Role of SERCA and Its Regulators

SERCA plays a key role in resequestering Ca^2+^ into the SR lumen during muscle relaxation. SERCA1 is expressed in fast-twitch skeletal muscles. SERCA2a is predominantly expressed in the heart and also found in slow-twitch muscles. SERCA3 is predominantly expressed in non-muscle tissues (Periasamy and Kalyanasundaram, 2007). In striated muscles, SERCA activity accounts for 70–90% of cytosolic Ca^2+^ removal (Periasamy and Kalyanasundaram, 2007).

It is now clear that the SERCA function is impaired in dystrophic muscles. SERCA activity can be reduced through several mechanisms, including (i) down-regulation of SERCA expression, (ii) posttranslational modification of SERCA protein, and (iii) differential expression and function of SERCA regulators. In the dystrophic myocardium of mouse models and human patients, SERCA2a levels remain unchanged (Voit et al., 2017; Wasala et al., 2020). In the *mdx* mouse, SERCA1a expression is increased in the spared intrinsic laryngeal and toe muscles but is reduced in the EDL muscle (Dowling et al., 2003; Ferretti et al., 2009). In the fast-twitch muscles of *mdx* and *mdx:utr*^−/−^ mice, SERCA2a expression is significantly increased, likely due to the increased number of slow-twitch fibers (Schneider et al., 2013; Voit et al., 2017). In the extensor carpi ulnaris muscles of the canine DMD model, SERCA2a levels are decreased while SERCA1 expression is unaltered (Voit et al., 2017). These findings suggest differential expression of SERCA isoforms in different dystrophic muscles. Given the different kinetic properties of SERCA1 and SERCA2a, it is likely the muscle- and species-specific changes in SERCA isoform expression represent compensatory alterations in different dystrophic muscles.

Irrespective of SERCA levels, SR Ca^2+^ uptake is significantly reduced in dystrophin-deficient cardiac and skeletal muscles, indicating decreased SERCA function (Schneider et al., 2013; Voit et al., 2017; Wasala et al., 2020; Mareedu et al., 2021). Oxidative posttranslational modification has been shown to play an important role in SERCA activity in non-dystrophic heart diseases (Lancel et al., 2010; Horakova et al., 2013). As dystrophic muscle undergoes oxidative stress (Kim et al., 2013), it is likely that similar posttranslational modifications can also affect SERCA function in DMD.

SERCA function is modulated by several small-molecular-weight membrane proteins including phospholamban (PLN), sarcolipin (SLN), myoregulin (MLN), and dwarf open reading frame (DWORF) (Bhupathy et al., 2007; Anderson et al., 2015; Nelson et al., 2016; Shaikh et al., 2016). PLN, SLN, and MLN are negative regulators, whereas DWORF is a positive regulator of SERCA. PLN is highly expressed in the ventricles (Babu et al., 2007). SLN is expressed in all skeletal muscle tissues in larger mammals; however, its expression is restricted to slow-twitch muscles in rodents (Babu et al., 2007). Whereas, in the hearts of both rodents and large mammals SLN is primarily expressed in atria and expressed at a low level in ventricles (Babu et al., 2007), MLN is primarily expressed in skeletal muscles (Anderson et al., 2015). DWORF is a positive regulator of SERCA and is predominantly expressed in the heart and slow-twitch muscles (Nelson et al., 2016). In addition to these peptides, recent studies suggest that small ubiquitin-like modifier type 1 (SUMO-1) also plays a critical role in cardiac SERCA dysfunction in the setting of heart failure (Kho et al., 2015).

The PLN levels are unaltered in dystrophic skeletal and cardiac muscles, whereas SLN is significantly up-regulated in the skeletal muscles and the ventricles of mouse and dog models and DMD patients (Voit et al., 2017). Little is known about the expression of MLN, DWORF, and SUMO-1 in dystrophin-deficient cardiac and skeletal muscles. It is worth pointing out that complete elimination of PLN exacerbates *mdx* cardiomyopathy (Law et al., 2018), whereas partial or complete elimination of SLN significantly reduces skeletal muscle disease and cardiomyopathy in mouse models of DMD (Voit et al., 2017; Mareedu et al., 2021).

### Role of Other SR Ca^2+^ Handling Proteins

The SR luminal resident proteins, including calsequestrin (CSQ), CSQ-like proteins (CLPs), histidine-rich Ca^2+^-binding protein (HRCBP), calreticulin, and sarcalumenin (SLM), play an important role in buffering luminal Ca^2+^ concentrations and regulating SR Ca^2+^ uptake and release (Beard et al., 2004; Arvanitis et al., 2011; Jiao et al., 2012). Among the various SR luminal proteins, CSQ is the major Ca^2+^-buffering protein. It is localized within the terminal cisternae of the SR in both cardiac and skeletal muscles. CSQ exists in two isoforms. CSQ1 is mainly expressed in skeletal muscles, whereas CSQ2 is expressed in cardiac and slow-twitch muscle (Murphy et al., 2009).

Increased levels of CSQ and calmodulin (CaM) have been reported in spared extraocular and intrinsic laryngeal muscles of *mdx* mice, whereas these proteins are at low levels in the diaphragm of *mdx* mice (Pertille et al., 2010). The same study also reported that CSQ level was significantly reduced in the soleus and sternomastoid muscles but unaltered in the tibialis anterior (TA) muscles, whereas CaM level decreased only in the TA muscle (Pertille et al., 2010). Collectively, the fiber-type composition of dystrophic and spared muscles in *mdx* mice could contribute to the altered levels of CSQ. There is a discrepancy in the protein levels of cardiac CSQ2 in the dystrophic myocardium. It has been shown that the levels of CSQ2 were drastically reduced in the heart of *mdx* mice (Lohan and Ohlendieck, 2004). However, we and others have shown that CSQ2 levels are unaltered in the hearts of *mdx* and *mdx:utr*^−/−^ mice (Pertille et al., 2010; Voit et al., 2017; Mareedu et al., 2021). Surprisingly, CSQ2 expression is aberrantly increased in the fast-twitch muscles of *mdx* and *mdx:utr*^−/−^ mice (Schneider et al., 2013; Voit et al., 2017). The exact reason for the increase of CSQ2 is not known but may likely be due to the fast- to slow-muscle fiber type switch in mouse models of DMD. The CLPs are significantly reduced in *mdx* skeletal muscle (Culligan et al., 2002). The expression of HRCBP is increased in the *mdx* heart (Lohan and Ohlendieck, 2004). The SLM expression is reduced in both skeletal and cardiac muscles in *mdx* mice (Lohan and Ohlendieck, 2004). Overall, changes in the SR luminal Ca^2+^-binding proteins support the concept of abnormal ${\text{Ca}}_{\text{i}}^{2+}$ cycling in dystrophic muscles. The differences in the expression levels of these proteins could reflect the severity of the muscle pathology, which varies between disease-free extraocular muscle, slow- and fast-twitch skeletal muscles, and cardiac muscles.

## Calcium Handling in Dystrophin-Deficient Smooth Muscles

Although the role of Ca^2+^ dysregulation has been extensively studied in dystrophin-deficient cardiac and skeletal muscle tissues, the implications of dystrophin deficiency in smooth muscles in DMD patients or animal models have not been adequately studied. Studies on the gastric and intestinal smooth muscles of *mdx* mice have shown impaired muscle relaxation, which has been attributed to impairment in ${\text{Ca}}_{\text{i}}^{2+}$ homeostasis (Mule et al., 1999; Mule and Serio, 2001). These studies show that in dystrophic colonic muscle, increased Ca^2+^ influx through L-type voltage-dependent channels is responsible for the sustained mechanical tone. A recent study showed that the enhanced stretch-induced ${\text{Ca}}_{\text{i}}^{2+}$ concentration in the vascular smooth muscle cells of *mdx* mice occurs through the activation of TRPC1, TRPC3, and TRPC6 channels (Lopez et al., 2020). Thus, focusing on the Ca^2+^ handling in dystrophic smooth muscle cells may have therapeutic implications.

## Mitochondria and Abnormal ${\text{Ca}}_{\text{i}}^{2+}$ Handling in DMD

**Figure 3:**
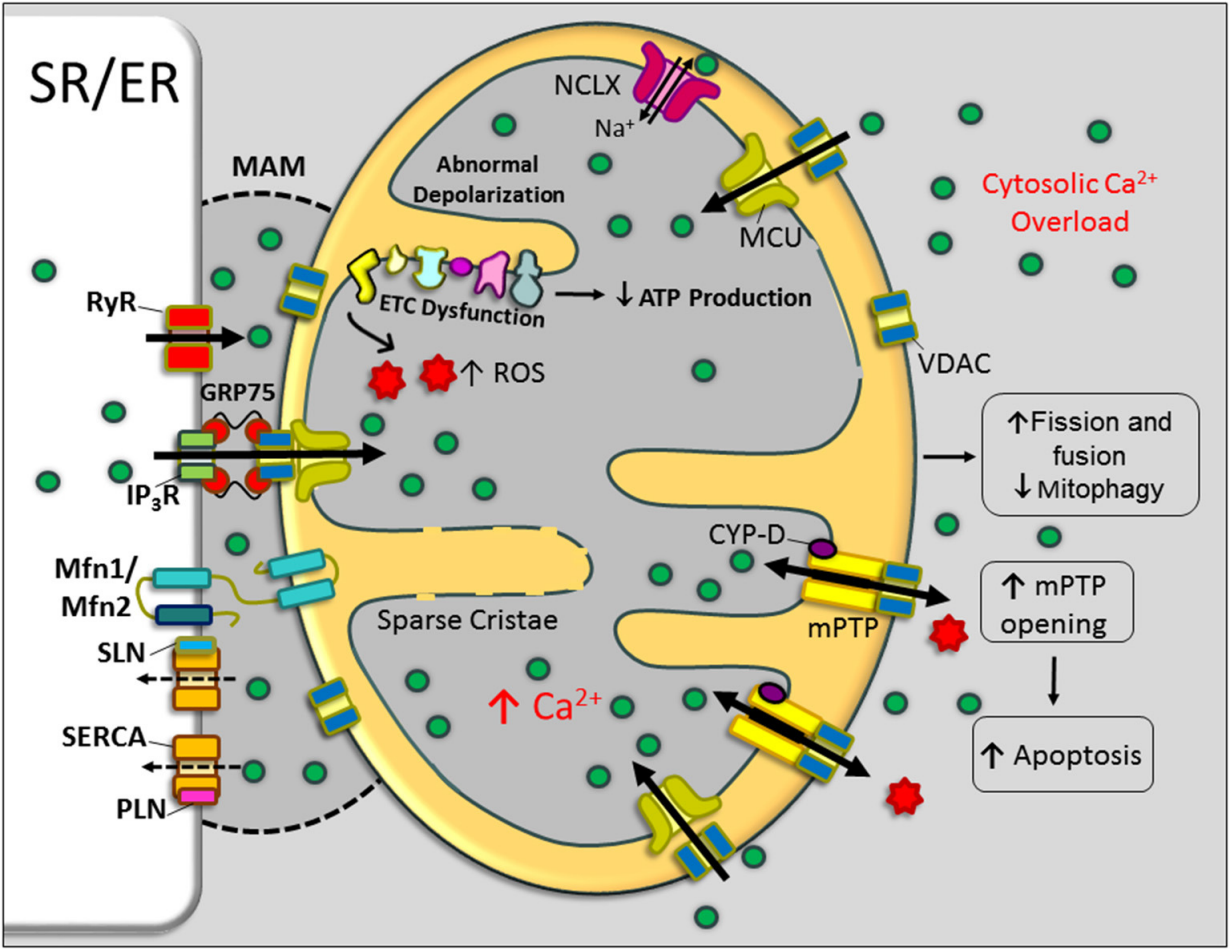
Mitochondrial dysfunction in DMD. Schematic representation of mitochondrial structural and functional alterations in dystrophin-deficient cardiac/skeletal muscle cells. An abnormal elevation of Ca^2+^ in the cytoplasm and mitochondria-associated membrane (MAM) region resulted in increased mitochondrial Ca^2+^ uptake and enhanced activation of mitochondrial permeability transition pore (mPTP) opening. These changes impair mitochondrial function and mitochondrial dynamics and contributing to the metabolic crisis. Currently, there is no experimental evidence for the role of the MAM region and mitochondrial Ca^2+^ uniporter (MCU) in the mitochondrial Ca^2+^ overload in DMD. Bold and broken arrows indicate the enhanced and decreased function of the Ca^2+^ channels, respectively. ER, endoplasmic reticulum; ETC, electron transport chain; GRP75, glucose-regulated protein 75; IP3R, inositol trisphosphate receptor; MFN, mitofusin; NCLX, Na^+^-Ca^2+^-Li^+^ exchanger; PLN, phospholamban; ROS, reactive oxygen species; RyR, ryanodine receptor; SERCA, sarcoplasmic/endoplasmic reticulum Ca^2+^ ATPase; SLN, sarcolipin; SR, sarcoplasmic reticulum; VDAC, voltage-dependent anion channel.

Mitochondria not only play a vital role in muscle bioenergetics but also contribute to Ca^2+^ homeostasis in striated muscles. Mitochondria store Ca^2+^ transiently (~10 nmol/mg of mitochondrial protein) (Finkel et al., 2015). Ca^2+^ levels in the mitochondria are an important determinant of mitochondrial function and cell survival (Williams et al., 2013; Finkel et al., 2015). When cytoplasmic Ca^2+^ levels are elevated, mitochondrial Ca^2+^ uptake is enhanced (Robert et al., 2001) as a compensatory mechanism to normalize the cytoplasmic Ca^2+^ concentration. Ca^2+^ uptake by mitochondria mainly occurs via the mitochondrial Ca^2+^ uniporter (MCU), whereas Ca^2+^ extrusion occurs through the mitochondrial Na^+^-Ca^2+^-Li^+^ exchanger (mtNCLX) (Pathak and Trebak, 2018). During conditions such as mitochondrial Ca^2+^ overload and mitochondrial dysfunction, the mitochondrial permeability transition pore (mPTP), a large channel in the inner mitochondrial membrane, also contributes to Ca^2+^ extrusion (Kwong and Molkentin, 2015). Here, we discuss the interplay between abnormal cytosolic and mitochondrial Ca^2+^ cycling and muscle pathogenesis in DMD (Figure 3).

### ${\text{Ca}}_{\text{i}}^{2+}$ Overload Contributes to Defective Mitochondrial Dynamics in DMD

Mitochondrial morphology change is a salient feature of dystrophic muscle. Specifically, the mitochondrial size is markedly reduced, the electron density is decreased, and the cristae become more sparse in dystrophic muscle (Pant et al., 2015; Kang et al., 2018; Moore et al., 2020). These changes are caused by increased mitochondrial fission and fusion cycles, referred to as mitochondrial dynamics, in dystrophic skeletal muscles (Pant et al., 2015). In the *mdx* heart, there is substantial structural damage to mitochondria and a significant reduction in ATP production. These changes are associated with decreased mitochondrial autophagy (mitophagy) due to decreased expression of proteins involved in the PINK1/PARKIN mitophagy pathway (Kang et al., 2018). These studies indicate that increased mitochondrial dynamics and decreased mitophagy could contribute to mitochondrial dysfunction and disease pathogenesis. It is important to note that mitochondrial abnormalities are reported in the *mdx* heart. These changes are associated with mitochondrial Ca^2+^ load and are preceded by cytosolic Ca^2+^ overload (Jung et al., 2008; Viola et al., 2013; Kyrychenko et al., 2015b). In support of this notion, our recent studies demonstrated that normalizing the ${\text{Ca}}_{\text{i}}^{2+}$ cycling improved the mitochondrial fission and fusion cycles in dystrophic myoblasts (Niranjan et al., 2019).

### Abnormal Mitochondrial Ca^2+^ Cycling, Mitochondrial Dysfunction, and Muscle Pathogenesis

Several studies have shown that mitochondria are potential targets of impaired Ca^2+^ homeostasis in muscular dystrophy. Increased mitochondrial Ca^2+^ content, impaired oxidative phosphorylation, increased ROS generation, decreased ATP production, aberrant mPTP opening, and abnormal mitochondrial depolarization have all been reported in cardiac and skeletal muscles of mouse models of DMD (Kuznetsov et al., 1998; Jung et al., 2008; Rybalka et al., 2014; Kyrychenko et al., 2015b; Kang et al., 2018). Similar to animal models, decreased oxidative phosphorylation has been reported in DMD patients (Sperl et al., 1997). Thus, cytosolic Ca^2+^ overload appears to directly contribute to mitochondrial dysfunction. In addition, mitochondrial dysfunction resulted in decreased ATP production, loss of membrane potential, and mPTP opening, which may further aggravate cytosolic Ca^2+^ overload in DMD muscle. In support of this, a recent study using the *Caenorhabditis elegans* as a model system showed that muscle damage in DMD could be a result of mitochondrial dysfunction, leading to cytoplasmic Ca^2+^ overload, which in turn activates various matrix metalloproteinase–mediated collagen degradation (Sudevan et al., 2019). Mitochondrial Ca^2+^ levels are elevated in aged dystrophic cardiomyocytes and are associated with the excessive opening of mPTP and loss of mitochondrial membrane potential (Kyrychenko et al., 2015b). Studies from Molkentin's laboratory have suggested that altered Ca^2+^ handling within the dystrophic muscle cells initiates cell necrosis, in part through the triggering of mPTP opening (Millay et al., 2008). These studies further showed treatment with the cyclophilin inhibitor Debio-025, which inhibits mPTP opening reduced mitochondrial swelling and necrosis in *mdx* mice (Millay et al., 2008). These studies suggest that inhibition of mPTP opening could be beneficial in DMD.

### Role of SR–Mitochondria Communication in Ca^2+^ Dysregulation in DMD

Emerging studies have revealed the presence of close contact between the ER and mitochondria called mitochondria-associated membranes (MAMs) (Figure 3). MAMs are specialized subdomains of the ER/SR and outer mitochondrial membrane (Garcia-Perez et al., 2011; Patergnani et al., 2011; Csordas et al., 2018). MAMs are critical for correct communication between the ER and mitochondria, particularly, the selective transmission of physiological and pathological Ca^2+^ signals from the ER to mitochondria (Patergnani et al., 2011). MAMs are enriched with voltage-dependent anion channels (VDACs), outer mitochondrial membrane proteins that aid in Ca^2+^ entry close to the junctional SR. In addition to VDAC, glucose-regulated protein 75 (molecular chaperone present on the ER membrane), mitofusin 2 (MFN2), and MFN1 are also involved in the physical interactions between ER/SR and mitochondria (Szabadkai et al., 2006; de Brito and Scorrano, 2008). MAMs also contain proteins involved in ER-associated lipid metabolism, Ca^2+^-handling proteins, and mitochondrial fission and fusion proteins (Szabadkai et al., 2006; de Brito and Scorrano, 2008; Flis and Daum, 2013; Vance, 2015).

The tight tether between ER/SR and mitochondrial membranes allows Ca^2+^ to be rapidly transferred. This design overcomes the low apparent Ca^2+^ affinity (*K*_d_ ~ 15–20 M) of the MCU (Rizzuto et al., 1998; Filippin et al., 2003). Many ER/SR-associated Ca^2+^-handling proteins, such as SERCA, inositol 1,4,5-trisphosphate receptor type 2 (IP3R2), RyR2, SERCA2, CSQ, and PLN, have been found enriched at MAMs, supporting the close correlation between this organellar intersection and Ca^2+^ regulation (Simpson and Russell, 1997; Simpson et al., 1997; Garcia-Perez et al., 2008; Patergnani et al., 2011; Chen et al., 2012; Raturi and Simmen, 2013). A recent study shows that pharmacological blockade or down-regulation of IP3R expression can restore mitochondrial Ca^2+^ levels, mitochondrial membrane potential, mitochondrial dynamics, and mitophagy in *mdx* muscles (Valladares et al., 2018). Another study showed that incubation of Sol8 myotubes expressing mini-dystrophin in cyclosporine A (CsA) normalized IP3R expression levels and aided in better cell survival (Mondin et al., 2009). These studies suggest that IP3R-associated Ca^2+^ dysregulation in the MAM region affects mitochondrial Ca^2+^ content and mitochondrial function in DMD. These findings lead to the speculation that altered expression levels of RyR, IP3R, and SERCA isoforms and their regulators in the MAM region could deeply affect the ${\text{Ca}}_{\text{i}}^{2+}$ signaling in this microdomain and thereby the cell metabolism and cell survival in DMD (Figure 3). A more detailed examination of MAM proteins in dystrophic muscle is needed to understand the mechanisms associated with Ca^2+^ mishandling in this microdomain and its contribution to DMD pathogenesis.

In summary, elevated cytoplasmic Ca^2+^ levels including increased Ca^2+^ levels in the MAM region increase mitochondrial Ca^2+^ load, which affects the mitochondrial structure, function, and mPTP opening. This further exacerbates cytosolic Ca^2+^ overload and subsequently contributes to DMD pathogenesis.

### Calcium, NOX, and ROS

Increasing evidence suggests functional crosstalk between Ca^2+^ and ROS signaling systems. ROS are generated both in cytosol and mitochondria by NOX and electron complex chain, respectively (Gorlach et al., 2015). ROS produced by NOX5 has been implicated in cardiovascular diseases, renal diseases, and cancer (Touyz et al., 2019). However, studies on the role of NOX isoforms in DMD are limited. In *mdx* mice, NOX2 was shown to be induced in skeletal muscle, whereas NOX4 was induced in the heart (Spurney et al., 2008). Nifedipine treatment, which reduces resting ${\text{Ca}}_{\text{i}}^{2+}$ concentrations, is associated with reduced expression of gp91phox/p47phox NOX2 subunits (Altamirano et al., 2013). The genetic down-regulation of NOX2 activity reduces Mn^2+^/Ca^2+^ influx across the dystrophin-deficient sarcolemma and restores autophagy and reduces muscle pathology in *mdx* mice (Pal et al., 2014; Loehr et al., 2016). These studies indicate the involvement of NOX2-driven oxidative stress in increased sarcolemmal Ca^2+^ influx in dystrophic muscles. NOX2-driven oxidative stress was also shown to be involved in dystrophic cardiomyopathy (Shirokova and Niggli, 2013). The expression of NOX2 is increased in the *mdx* heart along with increased superoxide production (Gonzalez et al., 2014). Furthermore, NOX2 inhibition by apocynin restored the Ca^2+^-handling properties, including the amplitude of Ca^2+^ transient and SR Ca^2+^ content in the dystrophic myocytes. In addition, NOX2 inhibition decreases the SR Ca^2+^ leak in *mdx* myocytes (Gonzalez et al., 2014). On the other hand, genetic inhibition of NOX2 production (by deleting the p47^phox^, a NOX2 regulatory subunit) failed to decrease RyR1 Ca^2+^ leak in *mdx* muscle fibers. These studies further show that NOX4 is activated in the NOX2 ROS-deficient *mdx* muscle and contributes to RyR1 leak (Cully and Rodney, 2020). Based on these findings, the authors proposed ROS-Ca^2+^ crosstalk, in which NOX4-ROS induces RyR1 Ca^2+^ leak and thereby increases the SR/T-tubular junctional Ca^2+^ concentration, which exacerbates NOX2 ROS and contributes to muscle pathology. A recent study shows the down-regulation of miR-448-3p in the *mdx* heart increased *Ncf1* expression, which encodes p47^phox^ (Kyrychenko et al., 2015a). These studies suggest the involvement of NOX2 in oxidative stress and enhanced Ca^2+^ signaling in dystrophic myocardium. In dystrophic myotubes, NOX inhibition abolished iPLA2 activity and reduced Ca^2+^ influx through stretch-activated and store-operated channels (Ismail et al., 2014). In addition, NOX inhibition in *mdx* muscle restored the force loss induced by eccentric contractions and reduced membrane damage (Ismail et al., 2014). Thus, NOX are appealing targets in developing new therapies for muscular dystrophy.

## Targeting Ca^2+^ Handling Proteins—A Therapeutic View

In light of the significant contribution of Ca^2+^ mishandling in DMD pathogenesis, strategies targeting Ca^2+^ handling may hold great promise to treat DMD. Preclinical studies in animal models have provided compelling evidence supporting these therapeutic modalities (Table 1).

**Table 1:** Therapies targeting dysregulated Ca^2+^ directly or indirectly.

**Drugs**	**Mechanism of action**	**Models used**	**Comments**	**References**
Verapamil, diltiazem, nifedipine	Ca^2+^-channel blockers	*mdx* mice	Shown benefit in a mouse model but failed to ameliorate the condition in clinical trials.	Matsumura et al., 2009; Altamirano et al., 2013; Spinazzola and Kunkel, 2016
Calpastatin	Calpain blocker	*mdx* mice, Canine model	Initially rescued the dystrophic phenotype in mice but C-101, a leupeptin-based drug was unsuccessful in the canine model	Spencer and Mellgren, 2002; Selsby et al., 2010; Childers et al., 2011
Streptomycin, spider venom	SAC channel blocker	*mdx* mice	Mitigate cytosolic Ca^2+^ rise	Yeung et al., 2005
Enalapril	ACE inhibitor	DMD patients	Improved cardiac function	Kwon et al., 2012
Carvedilol	β-Blocker	DMD patients	Improved cardiac function	Kwon et al., 2012
P-188 NF	Membrane sealant	*mdx* and *mdxutr^−/−^* mice, canine model	Improved cardiac and respiratory function	Yasuda et al., 2005; Townsend et al., 2010; Houang et al., 2015; Markham et al., 2015
Rycal	RyR-stabilizing compound	*mdx* mice, patient-derived cells	Attenuated SR Ca^2+^ leak and mitigate DMD phenotype	Capogrosso et al., 2018; Barthelemy et al., 2019
AAV.SERCA2a	Overexpression of SERCA2a	*mdx* mice and Canine model	Enhance SR Ca^2+^ uptake and ameliorate DMD	Goonasekera et al., 2011; Duan, 2015; Wasala et al., 2020
AAV.SERCA1	Overexpression of SERCA1	*mdx* mice	Enhance SERCA function and ameliorate DMD phenotype	Morine et al., 2010; Goonasekera et al., 2011; Mazala et al., 2015
AAV.SLN	Reducing the SLN expression levels	*mdx:utr^−/−^*mice	Enhance SERCA function and mitigate DMD	Voit et al., 2017
BGP-15	Inducer of Hsp-72	*mdx* and *mdx:utr^−/−^*mice	Improve muscle function by stabilizing SERCA function	Gehrig et al., 2012
Alisporivir	Cyclophilin D blocker	Zebrafish model	Enhanced mitochondrial function by preventing Ca^2+^ dependent mPTP opening	Schiavone et al., 2017

### Use of Non-specific Ca^2+^-Channel Blockers for the Treatment of DMD

Calcium accumulation has been demonstrated in skeletal muscle biopsies from fetal human DMD (Farini et al., 2016), suggesting Ca^2+^ abnormalities occur at the early onset of the disease. To determine the optimal time point for Ca^2+^ channel blocking to prevent the pathological onset of the disease, Jorgensen et al. treated *mdx* mice *in utero* with streptomycin, a non-specific Ca^2+^-channel blocker that inhibits stretch-activated and mechanosensitive ion channels (Jorgensen et al., 2011). Streptomycin treatment delayed the onset of dystrophic symptoms in the limb muscles in young *mdx* mice but did not prevent disease progression. In *mdx* mice, long-term treatment with streptomycin reduced limb-muscle pathology but worsened the diaphragm and cardiac pathology (Jorgensen et al., 2011). These studies argue that blocking Ca^2+^ channels before disease onset is not beneficial. On the other hand, streptomycin treatment after birth reduced creatine kinase activity in the diaphragm and sternomastoid muscles of *mdx* mice (Matsumura et al., 2011). These studies suggest that the differential effects of streptomycin in various dystrophic muscles may be associated with the differential expression of TRPC1 (Matsumura et al., 2011). Thus, care must be taken in the long-term clinical use of non-specific Ca^2+^-channel blockers in DMD patients.

### Blocking Ca^2+^ Entry Through Dystrophin-Deficient Sarcolemma

Membrane leaking has been suggested to contribute to cytosolic Ca^2+^ overload in dystrophic muscle. To address this issue, membrane sealants such as Poloxamer 188 and its derivatives have been developed to stabilize the sarcolemma. Preclinical studies in the murine and canine DMD model suggest that membrane sealants can improve cardiac function (Yasuda et al., 2005; Townsend et al., 2010; Houang et al., 2015; Markham et al., 2015). However, two studies failed to show protection in *mdx* mouse skeletal muscle (Quinlan et al., 2006; Terry et al., 2014). More studies are warranted to validate the therapeutic potency of P188 in preventing Ca^2+^ entry and ameliorating DMD.

Calcium can enter dystrophic muscle cells through the L-type Ca^2+^ channel. L-type Ca^2+^-channel blockers, such as diltiazem and verapamil, have been shown to reduce serum creatine levels and muscle necrosis in *mdx* mice (Matsumura et al., 2009). Similarly, nifedipine treatment restored cytosolic Ca^2+^ levels and improved muscle function in mouse models of DMD (Altamirano et al., 2013). Although these observations signify the therapeutic benefits of L-type Ca^2+^-channel blockers in animal models, several clinical trials have revealed no clinical benefit in DMD patients (Spinazzola and Kunkel, 2016). This could be due to many reasons, for example, differences in drug metabolisms and variation in drug dosing. Future studies are warranted to clarify the mechanisms underlying different outcomes in animal models and DMD patients and to develop creative strategies to improve the efficacy in human patients.

Targeting stretch-activated Ca^2+^ channels with streptomycin and spider venom toxin has been shown to attenuate ${\text{Ca}}_{\text{i}}^{2+}$ rise and muscle damage in *mdx* mice (Yeung et al., 2005). Pharmacological inhibition of TRPC1 and TRPC3 channels has been shown to reduce the Ca^2+^ entry into dystrophic skeletal myofibers and also improve their function (Vandebrouck et al., 2002). In *mdx* mice, overexpression of a dominant-negative mutant of the TRPV2 ion channel protects eccentric contraction-induced damage (Zanou et al., 2009). Thus, inhibiting TRP channels is a potential therapeutic strategy to prevent Ca^2+^ entry.

### Targeting Intracellular Ca^2+^ Signaling to Improve Cardiac Function

Angiotensin II or β-receptor–mediated signaling pathways, such as G-protein–coupled receptor pathways, modulate muscle contraction–relaxation via altered ${\text{Ca}}_{\text{i}}^{2+}$ handling. β_2_-adrenergic receptor–coupled pathway has been shown to attenuate skeletal muscle degeneration in DMD (Smith et al., 2002; Church et al., 2014; Silva et al., 2014). Treating albuterol, a β_2_ agonist, increased muscle strength in animal models but failed to improve muscle function in DMD patients (Skura et al., 2008). On the other hand, treating DMD patients with enalapril, an angiotensin-converting enzyme (ACE) inhibitor, or carvedilol, a β-blocker, improved the left ventricular systolic function without significant adverse effects in one patient study (Kwon et al., 2012).

### Preventing SR Ca^2+^ Leak

SR Ca^2+^ leak is a significant contributor to cytosolic Ca^2+^ overload in DMD. Rycal, a RyR-stabilizing compound, was found to attenuate muscle disease in *mdx* mice (Bellinger et al., 2009; Fauconnier et al., 2010; Capogrosso et al., 2018). Rycal is suggested to improve binding of calstabin to RyR and to prevent SR Ca^2+^ leak, thereby restoring ${\text{Ca}}_{\text{i}}^{2+}$ homeostasis (Capogrosso et al., 2018). A recent study showed that Rycal treatment bolsters antisense oligonucleotide-mediated exon skipping in patient-derived myotubes and induced pluripotent stem cell–derived diseased cardiomyocytes (Barthelemy et al., 2019). Although the therapeutic value of Rycal needs to be further validated, these studies suggest that targeting the RyR will boost treatment efficiency in DMD patients.

### Enhancing SR Ca^2+^ Uptake

Enhancing cytosolic Ca^2+^ removal is another powerful approach to restore the ${\text{Ca}}_{\text{i}}^{2+}$ homeostasis in dystrophic muscle. This can be achieved by overexpressing SERCA or by targeting its inhibitors to enhance SERCA activity. Several animal studies have shown the beneficial effects of SERCA overexpression in ameliorating muscular dystrophy in mice (Morine et al., 2010; Goonasekera et al., 2011; Shin et al., 2011; Mazala et al., 2015; Wasala et al., 2020). Transgenic overexpression of SERCA1 in skeletal muscles of *mdx* mice has been shown to enhance the EC coupling, improve Ca^2+^ removal from the cytosol, and attenuate dystrophic phenotypes such as fibrosis and increased serum creatine kinase levels (Goonasekera et al., 2011). Similarly, transgenic overexpression of SERCA1 is shown to ameliorate the structural damage and functional impairments of muscles from *mdx:utr*^−/−^ *mice* (Mazala et al., 2015). Adeno-associated virus (AAV)-mediated overexpression of SERCA1 in the diaphragm of *mdx* mice increased the proportion of type IIA fibers, reduced the percentage of centrally nucleated fibers, and attenuated the loss of force production following eccentric contractions (Morine et al., 2010). In contrast to SERCA1, SERCA2a is expressed in both skeletal muscle and the heart. We evaluated AAV SERCA2a gene therapy. Delivery to 12-month-old *mdx* mice significantly improved cardiac electrophysiology (Shin et al., 2011). A single intravenous injection in 3-month-old *mdx* mice significantly improves whole-body muscle performance and ameliorates fatal dilated cardiomyopathy in *mdx* mice up to 21 months of age (Wasala et al., 2020). The AAV.SERCA2a vector has been extensively studied in heart failure patients (Zsebo et al., 2014; Lyon et al., 2020). Lessons learned from these studies will help facilitate the translation of AAV.SERCA2a therapy in DMD patients.

Targeting SERCA regulators such as PLN and SLN also has therapeutic implications. Reducing PLN activity is shown to improve Ca^2+^ handling in animal models of heart failure (Kranias and Hajjar, 2012). Surprisingly, genetic PLN knockout worsened cardiac function in *mdx* mice (Law et al., 2018). It is yet unclear whether the partial reduction of PLN activity is beneficial in DMD. Unlike PLN, SLN up-regulation is a common molecular signature in all dystrophin-deficient muscles. Recent studies from our laboratory and others have shown that germline ablation of SLN ameliorates severe muscular dystrophy in a mouse model of DMD (Voit et al., 2017; Tanihata et al., 2018; Mareedu et al., 2019). Most strikingly, the loss of one SLN allele extended the lifespan of *mdx:utr*^−/−^ mice to 20 months. Also, our studies show that the reduction in SLN expression can improve muscle regeneration and prevent fiber-type transition in dystrophic muscles (Voit et al., 2017). Furthermore, AAV-mediated SLN reduction normalized ${\text{Ca}}_{\text{i}}^{2+}$ cycling and improved fusion and differentiation of dystrophin-deficient dog myoblasts (Niranjan et al., 2019). Our recent studies have demonstrated that reducing SLN expression is sufficient to restore cardiac SERCA function and ${\text{Ca}}_{\text{i}}^{2+}$ cycling and to prevent the development of cardiomyopathy in *mdx* mice throughout their lifespan (Mareedu et al., 2021). To translate these findings into a therapeutic strategy, we also knocked down SLN expression in 1-month-old *mdx:utr*^−/−^ mice via AAV-mediated RNA interference (Voit et al., 2017). This AAV treatment markedly reduces SLN expression, attenuates muscle pathology, and improves diaphragm, skeletal muscle, and cardiac function (Voit et al., 2017). In summary, these studies suggest that targeting SLN expression or function is a promising therapeutic strategy to improve SERCA function and ameliorate skeletal muscle disease and cardiomyopathy in DMD.

The therapeutic potential of targeting DWORF has been explored in animal models of heart failure. Cardiac-specific overexpression of DWORF enhances SERCA function, Ca^2+^ cycling, and contractility in mice (Nelson et al., 2016). In addition, overexpression of DWORF in the heart improves Ca^2+^ cycling, prevents pathological remodeling, and improves cardiac function in the muscle-specific LIM protein (MLP) knockout mouse model of dilated cardiomyopathy (Makarewich et al., 2018). A recent study shows that AAV-mediated overexpression of DWORF improved cardiac function in MLP knockout mice, as well as in the myocardial infarction model of heart failure (Makarewich et al., 2020). The expression levels and function of DWORF in dystrophic cardiac and skeletal muscle are yet to be studied. Nevertheless, based on the current findings, DWORF overexpression could be a therapeutic strategy to improve SERCA function and ameliorate muscular dystrophy and cardiomyopathy in DMD.

In addition to these strategies, improving SERCA function through other mechanisms has been shown to ameliorate muscular dystrophy in mice. Transgenic overexpression of heat shock protein 72 (Hsp72) or pharmacological induction of Hsp72 improves several pathological indices in *mdx* and *mdx:utr*^−/−^ mice, partly through the enhancement of SERCA function. These studies show that Hsp72 stabilizes the SERCA pump and increases its activity in dystrophic muscles (Gehrig et al., 2012). A recent study shows that blocking the insulin-like growth factor 2 receptor activates SERCA function and enhances the ${\text{Ca}}_{\text{i}}^{2+}$ removal in muscles of *mdx* mice (Bella et al., 2020).

### Reducing Mitochondrial Contribution to Cytosolic Ca^2+^ Overload

Improving mitochondrial function is another attractive therapeutic strategy for DMD. As discussed previously, Ca^2+^-dependent mitochondrial dysfunction could be due to the opening of mPTP (Briston et al., 2019). Thus, inhibiting cyclophilin, a key regulator of mPTP, could prevent mPTP opening and alleviate mitochondrial dysfunction in DMD. Although CsA treatment failed to improve muscle function in DMD patients (Kirschner et al., 2010), treatment with another cyclophilin inhibitor, Debio 025, which inhibits mPTP opening, has been shown to partially rescue the dystrophic phenotype in *mdx* mice (Reutenauer et al., 2008). Similarly, alisporivir, a derivative of CsA, has been shown to enhance the mitochondrial function in the zebrafish model of DMD (Schiavone et al., 2017). Further exploitation of these compounds and the identification of new compounds that prevent Ca^2+^-dependent mPTP opening provide an attractive therapeutic strategy to improve mitochondrial function and prevent muscle necrosis in DMD.

### Minimizing Downstream Events of Cytosolic Ca^2+^ Overload

Calpain activation leads to proteolytic damage in muscle cells. Overexpression of calpastatin, an endogenous inhibitor of calpain, significantly reduced muscle necrosis in *mdx* mice (Spencer and Mellgren, 2002). On the contrary, the use of C101, a leupeptin-based calpain inhibitor, failed to rescue phenotypes of DMD in animal models (Selsby et al., 2010; Childers et al., 2011). There is no direct study on the PLA_2_ inhibition in preventing muscle damage in DMD. However, corticosteroids, in clinical practice, suppress muscle inflammation possibly via PLA_2_ inhibition (Hoxha, 2019).

## Conclusions and Future Directions

Over the last several decades, there has been significant growth in our knowledge of the Ca^2+^ mishandling and Ca^2+^ homeostasis dysregulation in DMD. These findings have resulted in several highly promising and novel experimental therapies such as the use of the RyR stabilizer RyCal, SLN silencing, and viral vector–mediated SERCA overexpression. Yet, there are many unanswered questions and conflicting experimental results. ${\text{Ca}}_{\text{i}}^{2+}$ dysregulation is not only implicated in DMD but also involved in other muscular dystrophies, such as limb-girdle muscular dystrophy and laminopathies. Further understanding of the molecular mechanisms involved in Ca^2+^ mishandling may shed light on the secondary disease-causing mechanisms in various forms of muscular dystrophies. This understanding will help identify new therapeutic targets for the treatment of these diseases. Based on current knowledge, future studies may be directed to the following areas for a better understanding of targeting Ca^2+^ dysregulation:

1. *Mitochondria:* Calcium is a key regulator of mitochondrial function. Mounting evidence indicates that mitochondrial dysfunction plays an important role in disease pathogenesis in DMD. Exploring the molecular mechanisms including the MAM region that link the cytoplasmic Ca^2+^ load and mitochondrial Ca^2+^ mishandling may lead to the identification of potential therapeutic targets.
2. *SR Ca*^2+^ *reuptake:* Based on our recent studies, enhancing SERCA2a expression or activity (through reducing SERCA inhibitors) appears to be a promising strategy to treat both cardiac and skeletal muscle defects in DMD. Therefore, exploring new strategies to improve SERCA function, developing new AAV vectors with improved tropism for both muscle and heart, and identifying small molecules that activate endogenous SERCA pumps will be useful.
3. *The use of large animal models:* Many Ca^2+^-targeted therapies were performed in mouse models of DMD. These findings need to be verified in large animal models before moving to human trials.
4. *The effects of dystrophin restoration on Ca*^2+^ *handling:* It is unknown whether partial dystrophin restoration alleviates Ca^2+^-handling defects in dystrophic muscles. This information is not available and needs to be tested. This will help to design a combination therapy of dystrophin restoration with improved Ca^2+^ handling.

## Disclosure

DD is a member of the scientific advisory board for Solid Biosciences and equity holders of Solid Biosciences. The Duan laboratory has received research supports unrelated to this project from Solid Biosciences and Edgewise Therapeutics in the past 3 years. SM, EM, and GB have no conflicts of interest, financial or otherwise. The content of this manuscript is solely the responsibility of the authors and does not necessarily represent the official views of the National Institutes of Health.

## Author Contributions

All authors discussed, wrote, and commented on the manuscript at all stages.

## Conflict of Interest

The authors declare that the research was conducted in the absence of any commercial or financial relationships that could be construed as a potential conflict of interest.
